# Haemorrhagic Lumbar Juxtafacet Cyst with Ligamentum Flavum Involvement

**DOI:** 10.1155/2014/126067

**Published:** 2014-12-14

**Authors:** Finn Ghent, Trent Davidson, Ralph Jasper Mobbs

**Affiliations:** ^1^Neuro Spine Clinic, Prince of Wales Private Hospital, Randwick, Sydney, NSW 2031, Australia; ^2^Department of Pathology, Prince of Wales Hospital, Randwick, Sydney, NSW 2031, Australia; ^3^School of Medical Sciences, University of New South Wales, Sydney, NSW 2031, Australia

## Abstract

Juxtafacet cysts are an uncommon cause of radiculopathy. They occur most frequently in the lumbar region, and their distribution across the spine correlates with mobility. Haemorrhagic complications are rare and may occur in the absence of any provocation, although there is some association with anticoagulation and trauma. We present a case of acute radiculopathy due to an L5/S1 juxtafacet cyst with unprovoked haemorrhage which was found to extend into ligamentum flavum. The patient underwent uncomplicated microscope assisted decompression with excellent results. The demographics, presentation, aetiology, and management of juxtafacet cysts are discussed.

## 1. Case Report

A 77-year-old female was referred with a 2-month history of sudden onset left leg pain fitting an S1 radiculopathy. There was a long history of mild lower back pain. There was no history of trauma. Her only medical history was deep venous thrombosis; however, the patient was not on anticoagulation therapy when her symptoms developed. On examination, there was no weakness, the left Achilles reflex was absent, and there was diminished sensation fitting the left S1 dermatome.

**Figure 1:**
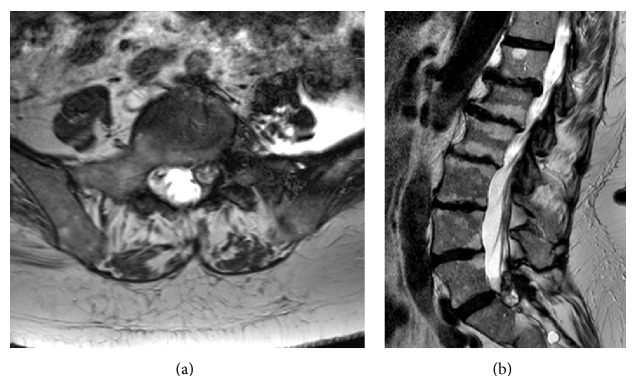
Axial (a) and sagittal (b) T2 weighted images showing the cyst with associated blood products at the left L5/S1 level.

Magnetic resonance imaging (MRI) revealed a cystic lesion adjacent to the left L5/S1 facet joint, as well as mixed intensity material extending into the surrounding tissues (Figure 1). 

**Figure 2:**
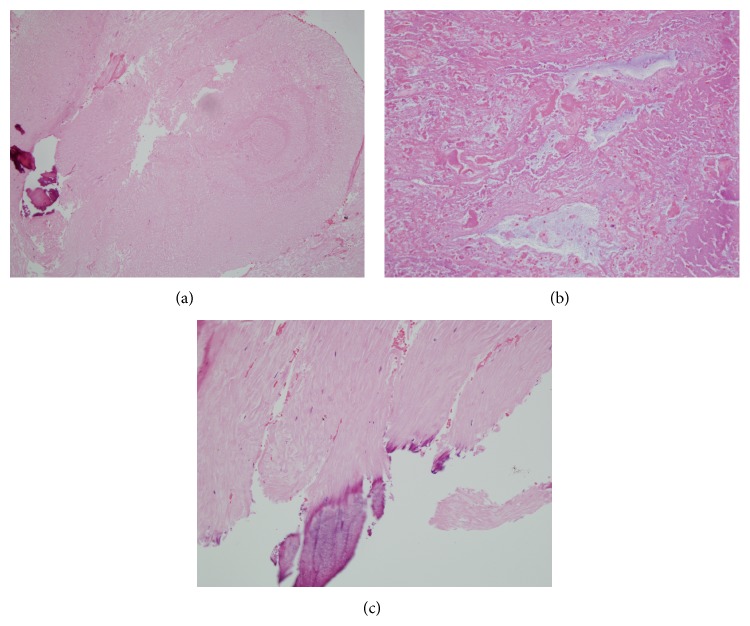
Low power haematoxylin and eosin (H&E) stain (a) showing fibroelastic tissue with patchy myxohyaline degenerative change. Fragments of debris and myxoid material, consistent with degenerative cyst contents (b). Fibroelastic tissue showing dystrophic calcification (c).

*Operation.* The left L5/S1 interspace was approached, and under microscope guidance a microlaminotomy was performed. A cyst capsule with haemorrhage extending into the ligamentum flavum was excised. The L5 and S1 nerve roots were decompressed. The patient recovered uneventfully. Histopathologic findings included fragments of ligamentum flavum, abundant myxohyaline degenerative material, haematoma, fibrofatty tissue, and dystrophic calcification. Slides of the cyst wall and degenerative contents are shown in Figure 2. No synovium was identified, and no malignant features were noted.

## 2. Discussion

First documented in the knee by Baker during the 1880s, joint associated cysts were recognised as a cause of nerve root compression in 1950 [1–3]. Mean age at presentation with juxtafacet cysts is 66 years (range 28–94 years), and imaging studies indicate that they are present in 0.5-0.6% of the population [4–6]. The most common presenting symptom is radiculopathy (62–98%), followed by back pain (53–79%) and neurogenic claudication (7–24%) [7, 8].

The distribution of juxtafacet cysts correlates with mobility and incidence of degenerative changes, with over 50% occurring at L4/5 and the remainder mostly found at L5/S1 and L3/4 [9–11]. They are uncommon in the cervical spine and even rarer in the thoracic spine. Coincident spondylolisthesis is seen in 43% of patients, osteoarthritis in 41%, and degenerative disc disease in 13% [7]. These associations are found consistently throughout the literature, implicating degenerative disease and instability in cyst pathogenesis.

Haemorrhagic changes are rare; less than 10% of juxtafacet cysts are found to have haemorrhagic complications [11–16]. No clear risk factor has been identified. The role of trauma provoking bleeding from the rich synovial vasculature has been suggested by Xu et al. [12] who identified a clear history of physical trauma in 8 of 30 patients. They also suggest repetitive microtrauma, unrecognized by the patient, as another potential cause. Two cases have identified anticoagulation as a precipitant [17, 18]. There was no identifiable precipitant in the case we have presented.

Christophis et al. [8] assessed cyst origin intraoperatively in 58 cases. They suggest juxtafacet cysts may originate from three places: the facet joint, ligamentum flavum, and posterior longitudinal ligament (PLL). Only cysts originating from the facet joint can histologically be true synovial cysts, making others pseudocysts, or synovial cysts where the lining has degenerated. The pathologic finding of fibroelastic cyst wall and myxohyaline degeneration and the intraoperative finding of hemorrhage extending into the ligamentum flavum are consistent with facet origin but leave open the possibility that this case may represent a pseudocyst originating from the flavum.

Owing to scarcity, there are no prospective randomized trials for the management of juxtafacet cysts. Expert consensus favours surgical management with laminectomy or laminotomy and cyst excision where they cause neurologic symptoms or intractable back pain. Haemorrhage does not affect management but may alter symptoms significantly and acutely. Conservative management with analgesics, braces, physiotherapy, steroid injections, and aspiration have been trialed, with results favouring surgical intervention [8, 11, 19].
